# Epstein Barr Virus-Induced 3 (EBI3) Together with IL-12 Negatively Regulates T Helper 17-Mediated Immunity to *Listeria monocytogenes* Infection

**DOI:** 10.1371/journal.ppat.1003628

**Published:** 2013-09-19

**Authors:** Yeonseok Chung, Tomohide Yamazaki, Byung-Seok Kim, Yongliang Zhang, Joseph M. Reynolds, Gustavo J. Martinez, Seon Hee Chang, Hoyong Lim, Mark Birkenbach, Chen Dong

**Affiliations:** 1 Department of Immunology and Center for Inflammation and Cancer, The University of Texas MD Anderson Cancer Center, Houston, Texas, United States of America; 2 Center for Immunology and Autoimmune Diseases, Institute of Molecular Medicine, The University of Texas Medical School at Houston, Houston, Texas, United States of America; 3 The Graduate School of Biomedical Sciences, The University of Texas Health Science Center at Houston, Houston, Texas, United States of America; 4 Department of Medicine, Section of Rheumatology, Temple University, Philadelphia, Pennsylvania, United States of America; University of Pittsburgh, United States of America

## Abstract

Although the protective functions by T helper 17 (Th17) cytokines against extracellular bacterial and fungal infection have been well documented, their importance against intracellular bacterial infection remains unclear. Here, we investigated the contribution of Th17 responses to host defense against intracellular bacteria *Listeria monocytogenes* and found that Th17 cell generation was suppressed in this model. Unexpectedly, mice lacking both p35 and EBI3 cleared *L. monocytogenes* as efficiently as wild-type mice, whereas p35-deficient mice failed to do so. Furthermore, both innate cells and pathogen-specific T cells from double-deficient mice produced significantly higher IL-17 and IL-22 compared to wild-type mice. The bacterial burden in the liver of double-deficient mice treated with anti-IL-17 was significantly increased compared to those receiving a control Ab. Transfer of Th17 cells specific for *listeriolysin O* as well as administration of IL-17 and IL-22 significantly suppressed bacterial growth in p35-deficient mice, indicating the critical contribution of Th17 responses to host defense against the intracellular pathogen in the absence of IL-12 and proper Th1 responses. Our findings unveil a novel immune evasion mechanism whereby the intracellular bacteria exploit IL-27EBI3 to suppress Th17-mediated protective immunity.

## Author Summary

There is a considerable gap in our understanding of how pathogenic intracellular bacteria escape innate and adaptive host immunity. Production of IL-12, and subsequently IFNγ, upon infection triggers host immunity that prevents early dissemination of pathogenic intracellular pathogens. This is evident in observing the increased susceptibility of patients with deficiencies in IL-12, IFNγ, or their receptors to pathogenic intracellular bacteria such as *Mycobacterium tuberculosis* and *Listeria monocytogenes* (Lm). Paradoxically, the regulation of host defense by other members of the IL-12 family is poorly understood. Through the use of an animal model of Lm infection, we show that mice lacking IL-27EBI3 were resistant to Lm infection, even in the absence of IL-12. Neutralization and adoptive transfer studies showed that this protection was mediated through IL-17, IL-22 and Th17 responses. Thus our results identify IL-27EBI3 as a critical mechanism for immune escape by Lm in the absence of IL-12-mediated protective immunity. Furthermore, our work suggests that targeting IL-27EBI3 may represent a novel strategy for the treatment of bacterial infection in individuals lacking proper IL-12 responses.

## Introduction

The generation of pathogen-specific T cell responses is essential for the clearance of infectious agents. This involves the differentiation of naïve T cells into distinct pathogen-specific helper T cell lineages in a process that largely depends on the cytokine milieu created by innate immune cells upon their activation. Among these innate cytokines, the IL-12 family plays a pivotal role during the differentiation of helper T cells by promoting or inhibiting the lineage program of Th1 or Th17 cells. IL-12 and Th1 responses mediate protective immunity against intracellular pathogens such as *Mycobacterium tuberculosis, Francisella tularemia*, and *Listeria monocytogenes* [1], [2]. Conversely, the production of IL-23 and the generation of Th17 responses are thought to mediate host defense against extracellular bacteria such as *Staphylococcus aureus, Klebsiella pneumoniae*, and *Citrobacter rodentum* [3], [4], [5], [6], as well as fungi such as *Candida albicans* and *Pneumocystis carnii* [7], [8]. The function of Th17 cells following intracellular bacterial infection is less clear.

The IL-12 gene family consists of p35, p40, p19, p28 and Epstein-Barr virus-induced 3 (EBI3). Different combination of two gene-products from this family results in the production of four cytokines: IL-12 (p35/p40), IL-23 (p19/p40), IL-27 (p28/EBI3) and IL-35 (p35/EBI3) [9], [10]. IL-12, IL-23 and IL-27 are produced by antigen-presenting cells such as dendritic cells (DC) and macrophages, whereas IL-35 is primarily produced by regulatory T cells [9], [10]. IL-12 is essential for promoting IFNγ production by innate cells such as NK and NKT cells following viral and bacterial infections. The IL-12 family also impacts adaptive T cell responses where IL-12 promotes Th1 generation and IL-23 promotes Th17 cells. IL-27 is thought to mediate the early phase of Th1 responses [11]. For instance, mice deficient in IL-27Rα exhibit reduced Th1 responses following infection with intracellular pathogens such as *Listeria monocytogenes* and *Leishmania major* [12], [13]. In contrast, others have shown that the IL-27 receptor signal is not required for Th1 polarization but rather inhibits IFNγ production by CD4^+^ T cells in an animal model of *Toxoplasma gondii* infection [14]. IL-27 has also been shown to suppress Th17 differentiation and Th17-mediated tissue inflammation [15], [16], probably by inducing the expression of PD-L1 on T cells [17]. More recently, it has been demonstrated that IL-27 drives the differentiation of IL-10 producing CD4^+^ T cells [18], [19], [20], suggesting anti-inflammatory function of this cytokine. Thus, IL-12 family of cytokines are involved in complex and often opposing roles in the development of helper T cell responses during infection and inflammation.

*Listeria monocytogenes* (Lm) is a Gram-positive, intracellular bacterium that can cause meningitis and encephalitis in immune-compromised individuals as well as reproductive issue in pregnant women [21]. The host defense against Lm involves a complex network of innate and adaptive immune cells. Following infection, Lm promptly triggers a series of innate immune cell activation where IFNγ produced mainly by natural killer (NK) cells contributes to initial resistance then triggers the induction of TNF-α and iNOS-producing dendritic cells (Tip-DC) that can control bacterial growth *in vivo*. In addition, neutrophils and macrophages are recruited and mediate killing of the intracellular pathogen. Finally, pathogen specific CD4^+^ T cells and CD8^+^ T cells are generated and mediate efficient bacterial clearance and recall responses to the pathogen [21]. γδ T cells may also be involved in an innate capacity as mice deficient in γδ T cells are more susceptible to the Lm infection [22]. In this regard, a recent study showed that IL-23 mediated activation of IL-17-producing γδ T cells can contribute the resistance against Lm infection [23], [24].

The importance of Th17 responses in the host defense against extracellular pathogens has been well described, however, whether Th17 cells and Th17 cytokines play a role against intracellular pathogen remains unclear. In addition, no study to date has fully addressed the relative contribution of IL-12 family cytokines following intracellular bacterial infection. To address these issues, we investigated anti-*Listeria* immunity in mice deficient in IL-12p35, IL-27EBI3, or both. Unexpectedly, our findings uncovered a dominant negative regulatory role of IL-27EBI3 in the protective immunity to Lm, especially in the absence of IL-12p35. The function of EBI3 was, at least in part, mediated by inhibiting the production of Th17 cytokines.

## Results

### Innate and helper T cell responses against *Listeria monocytogenes* infection

**Figure 1:**
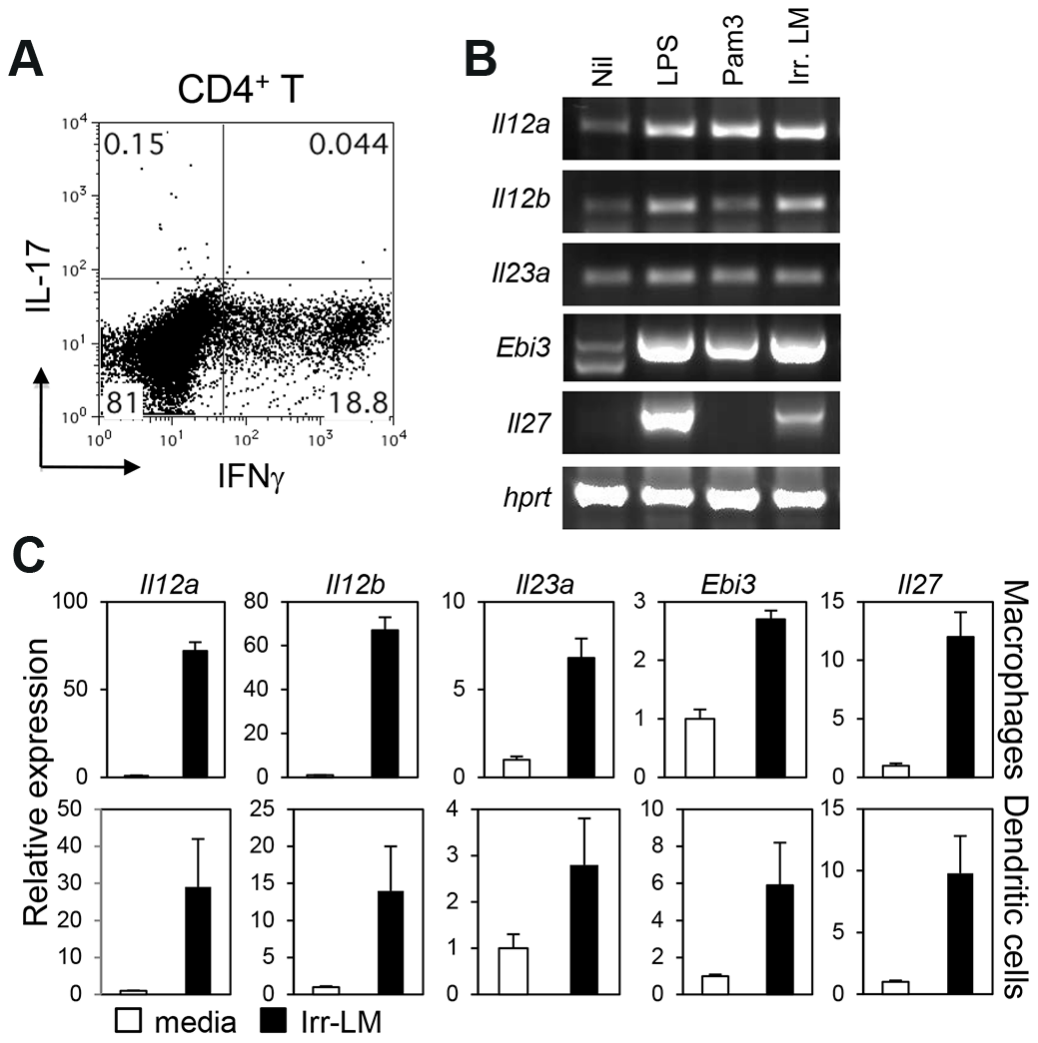
CD4^+^ T cell responses and the induction of IL-12 family genes after infection with *L. monotytogenes*. *A*, C57BL/6 mice were intravenously infected with 2.5×10^4^ Lm-Ova on day 0, and splenocytes were obtained on day 7. CD4^+^ T cells expressing IFNγ and IL-17 were measured by intracellular staining after stimulation with LLO_190–201_. *B and C*, Bone marrow-derived DC or macrophages were stimulated with LPS, Pam_3_CSK_4_, or irradiated Lm-Ova for four hours. Cells were harvested and analyzed for the mRNA expression of IL-12 family genes by using RT-PCR (*B*, bone marrow-derived DC), or quantitative real-time PCR analysis (*C*). Values are mean ± SD. Data shown represent two independent experiments.

Systemic infection with Lm is known to induce pathogen-specific Th1 cells. To examine if pathogen-specific Th17 cells are also generated during infection, we intravenously infected C57BL/6 mice with Lm expressing ovalbumin (Lm-Ova) [25], and examined the expression of IL-17 and IFNγ by splenic CD4^+^ T cells after restimulation with an Lm-specific, MHC II-restricted peptide (listeriolysin O (LLO)_190–201_). As expected, intravenous infection with live Lm-Ova induced a high percentage of IFNγ-producing CD4^+^ T cells (Figure 1A). By contrast, very few CD4^+^ T cells expressed IL-17 in the spleens of the infected mice.

Among the IL-12 family cytokines, IL-23 mediates Th17 immunity while IL-12 and IL-27 induce Th1 and suppress Th17 responses. To determine if the Lm dominant Th1 responses were due to a preferential induction of IL-12 and IL-27, we examined the induction of IL-12 family genes in dendritic cells and macrophages stimulated with lethally irradiated Lm. Importantly, irradiation induces the inactivation of Lm without affecting adjuvanticity and immunogenicity [26]. Stimulation of bone marrow-derived dendritic cells or macrophages with irradiated Lm induced the expression of *Il12a* (encoding IL-12p35), *Il12b* (encoding IL-12/IL23p40), *Il23a* (encoding IL-23p19), *Ebi3* (encoding IL-27EBI3) and *Il27* (encoding IL-27p28) as efficiently as LPS stimulation (Figure 1B & C). Together, these data demonstrate that while all genes in the IL-12 family were induced upon Lm encounter, only Th1 immunity was induced after systemic infection with Lm-Ova *in vivo*.

### Increased *Listeria*-specific Th17 responses in p35^−/−^EBI3^−/−^ mice

**Figure 2:**
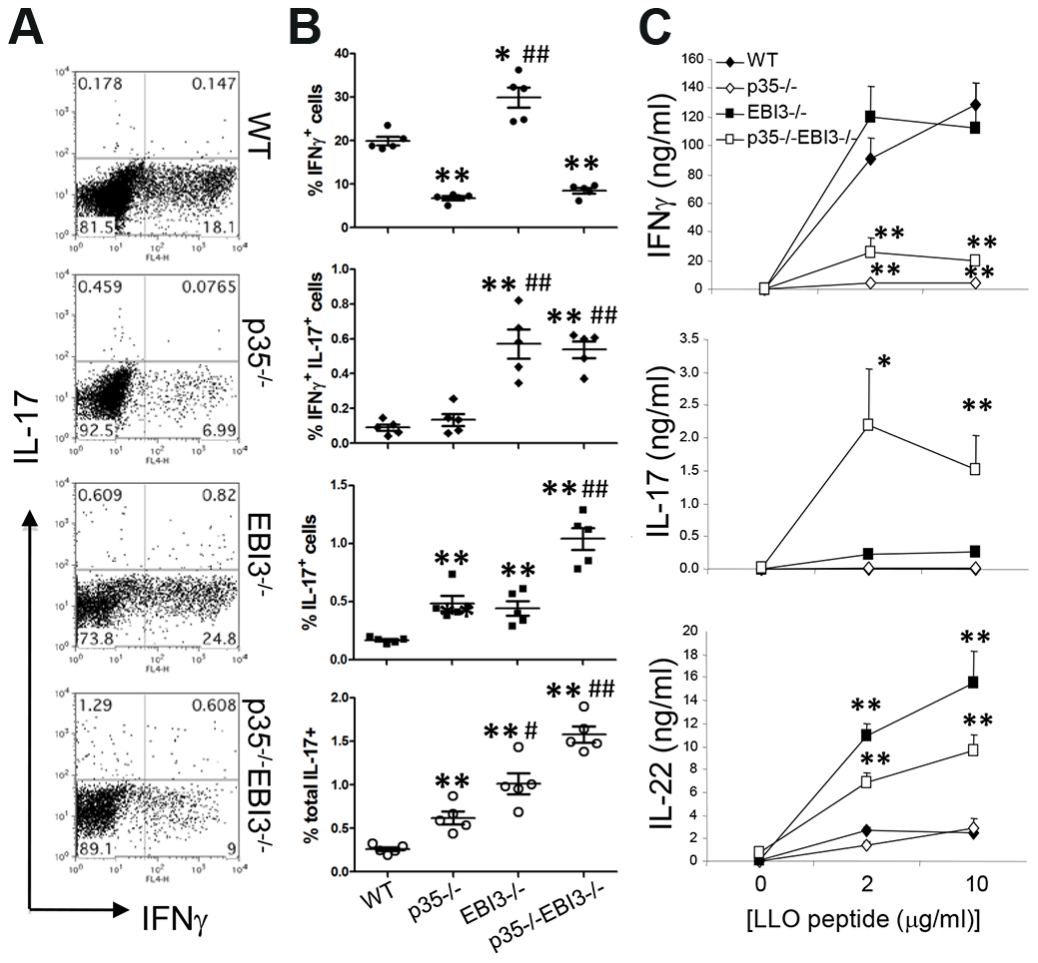
Increased pathogen-specific Th17 responses in the absence of IL-12p35 and IL-27EBI3. C57BL/6 (WT) or the indicated strains of mice (n = 5 per group) were intravenously infected with 2.5×10^4^ Lm-Ova on day 0. Seven days later, lymphoid cells from the spleen were obtained and CD4^+^ T cells expressing IFNγ and IL-17 were measured by intracellular staining after stimulation with LLO_190–201_ *(A and B)*. The lymphoid cells from the spleen were stimulated with LLO_190–201_ peptide for three days, and the concentrations of IFNγ, IL-17 and IL-22 in the supernatant were measured by ELISA *(C)*. Bars in B are mean ± SEM. Values in *C* are mean ± SEM. Data shown are representative of two independent experiments. *,p<0.05; **,p<0.01 in comparison with WT group. #,p<0.05; ##,p<0.01 in comparison with p35^−/−^ group.

We next sought to address whether the lack of pathogen-specific Th17 immunity in wild-type mice after Lm-Ova infection was due to IL-12 and IL-27. To analyze the relative contribution of IL-12p35 and IL-27EBI3, we first crossed p35^−/−^ mice with EBI3^−/−^ to generate p35^−/−^ EBI3^−/−^ mice. Wild-type, p35^−/−^, EBI3^−/−^, or p35^−/−^EBI3^−/−^ mice were then systemically infected with Lm-Ova via the intravenous route. Seven days later, we restimulated splenocytes from the infected mice with LLO_190–201_ to measure pathogen-specific CD4^+^ T cell responses. As expected, we observed high percentages of IFNγ-producing CD4^+^ T cells (∼20%), while few CD4^+^ T cells produced IL-17 in the wild-type mice (<0.5%) (Figure 2A & B). Compared with wild-type mice, the production of IFNγ by LLO-specific CD4^+^ T cells was greatly diminished in p35^−/−^ mice. Notably, although the IL-27 may be an inducer of Th1 responses [12], [13], we did not observe any defect in the percentage of IFNγ-producing CD4^+^ T cells in EBI3^−/−^ mice (Figure 2A & B). Instead, we observed that the frequency of IL-17-producing CD4^+^ T cells in the EBI3-deficient mice was significantly higher than those of wild-type mice, likely due to the increased population producing both IFNγ and IL-17 among CD4^+^ T cells (Figure 2A & B). Notably, compared with p35^−/−^ and EBI3^−/−^ mice, p35^−/−^EBI3^−/−^ mice exhibited a significantly increased frequency of IL-17^+^IFNγ^−^ CD4^+^ T cells (Figure 2A & B). Consequently, the production of IL-17 and IL-22 by Lm-specific CD4^+^ T cells was far higher in the p35^−/−^EBI3^−/−^ mice compared to wild-type mice (Figure 2C). p35^−/−^ and EBI3^−/−^ mice both showed a slight increase in the frequency of IL-17^+^ CD4^+^ T cells, however, the amounts of IL-17 produced after antigen restimulation were far less than that of p35^−/−^EBI3^−/−^ mice. Thus, p35^−/−^EBI3^−/−^ mice exhibited diminished Th1 and enhanced Th17 responses to Lm-Ova infection, indicating that IL-27EBI3 and IL-12p35 cooperatively suppress the generation of pathogen-specific Th17 cells after infection.

### Activation of CD8^+^ T cells and innate cells in the absence of IL-12p35 and/or IL-27EBI3 following *L. monocytogenes* infection

**Figure 3:**
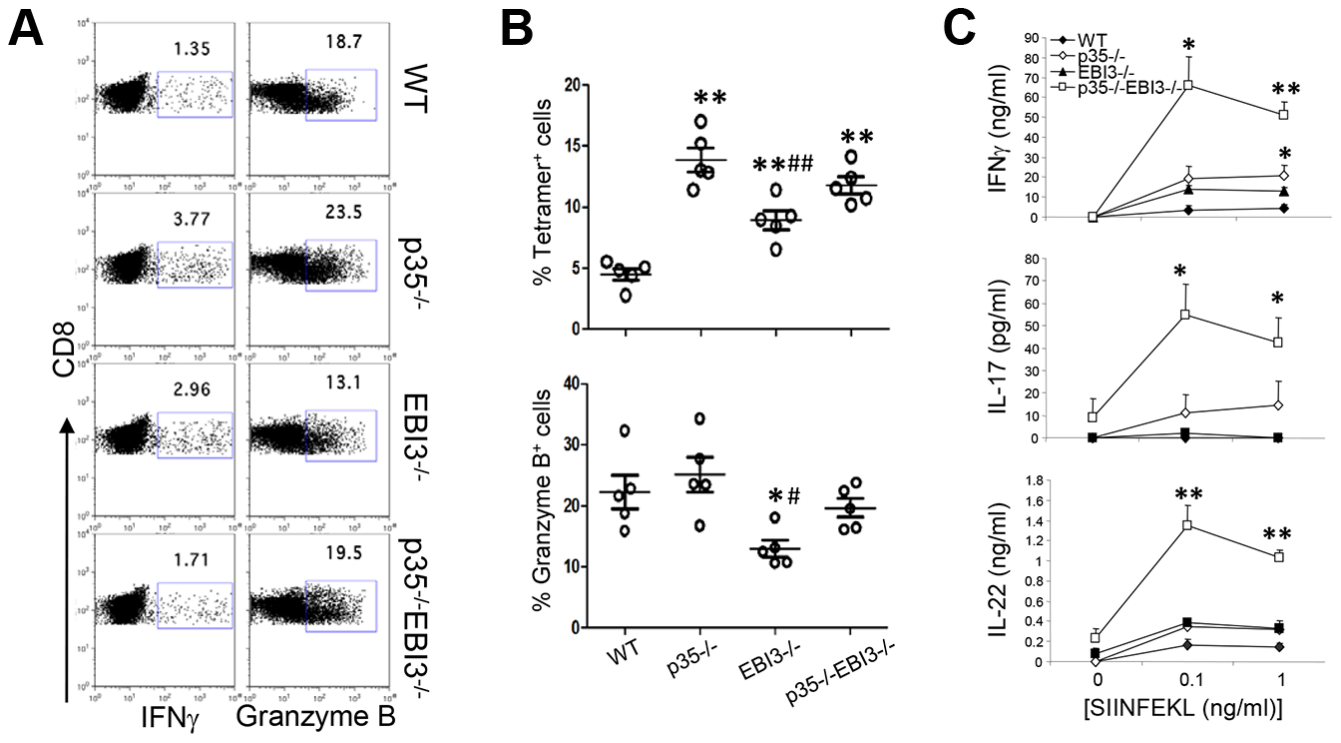
Pathogen-specific CD8^+^ T cell responses in the absence of IL-12p35 and IL-27EBI3. C57BL/6 (WT) or the indicated strains of mice (n = 5 per group) were intravenously infected with 2.5×10^4^ Lm-Ova on day 0. Seven days later, lymphoid cells from the spleen were obtained and CD8^+^ T cells expressing IFNγ and granzyme B were measured by intracellular staining after stimulation with SIINFEKL *(A and B)*. CD8^+^ T cells expressing T cell receptors specific to Ova were analyzed by staining with SIINFEKL-loaded K^b^ tetramer (*B*). The lymphoid cells from the spleen were stimulated with SIINFEKL peptide for three days, and the concentrations of IFNγ, IL-17 and IL-22 in the supernatant were measured by ELISA *(C)*. Bars in B are mean ± SEM. Values in *C* are mean ± SEM. Data shown are representative of two independent experiments. *,p<0.05; **,p<0.01 in comparison with WT group. #,p<0.05; ##,p<0.01 in comparison with p35^−/−^ group.

To measure the pathogen-specific CD8^+^ T cell responses to Lm-Ova, we restimulated splenocytes from infected mice with SIINFEKL peptide. CD8^+^ T cells derived from p35^−/−^ mice and EBI3^−/−^ mice exhibited similar or higher percentages of IFNγ compared to wild-type T cells (Figure 3A). Moreover, the percentages of Ova-specific MHC I tetramer-positive CD8^+^ T cells were significantly higher in p35^−/−^, EBI3^−/−^, and p35^−/−^EBI3^−/−^ mice compared to wild-type mice (Figure 3B). The frequencies of CD8^+^ T cells expressing granzyme B were comparable among wild-type, p35^−/−^, and p35^−/−^EBI3^−/−^ mice while decreased in EBI3^−/−^ mice (Figure 3A & B). Hence, the generation of pathogen-specific CD8^+^ T cells is largely independent of p35 and EBI3. These results are consistent, in part, with a previous study showing that IL-12 is not required for IFNγ production but rather inhibits the generation of memory CD8^+^ T cells [27]. By contrast, we observed that the amounts of IL-17 and IL-22 produced by CD8^+^ T cells were remarkably higher in p35^−/−^EBI3^−/−^ mice than those in the wild-type (Figure 3C). Hence, in the absence of IL-12p35 and IL-27EBI3, systemic Lm-Ova infection triggers increased production of IL-17 and IL-22 by pathogen-specific CD8^+^ T cells.

**Figure 4:**
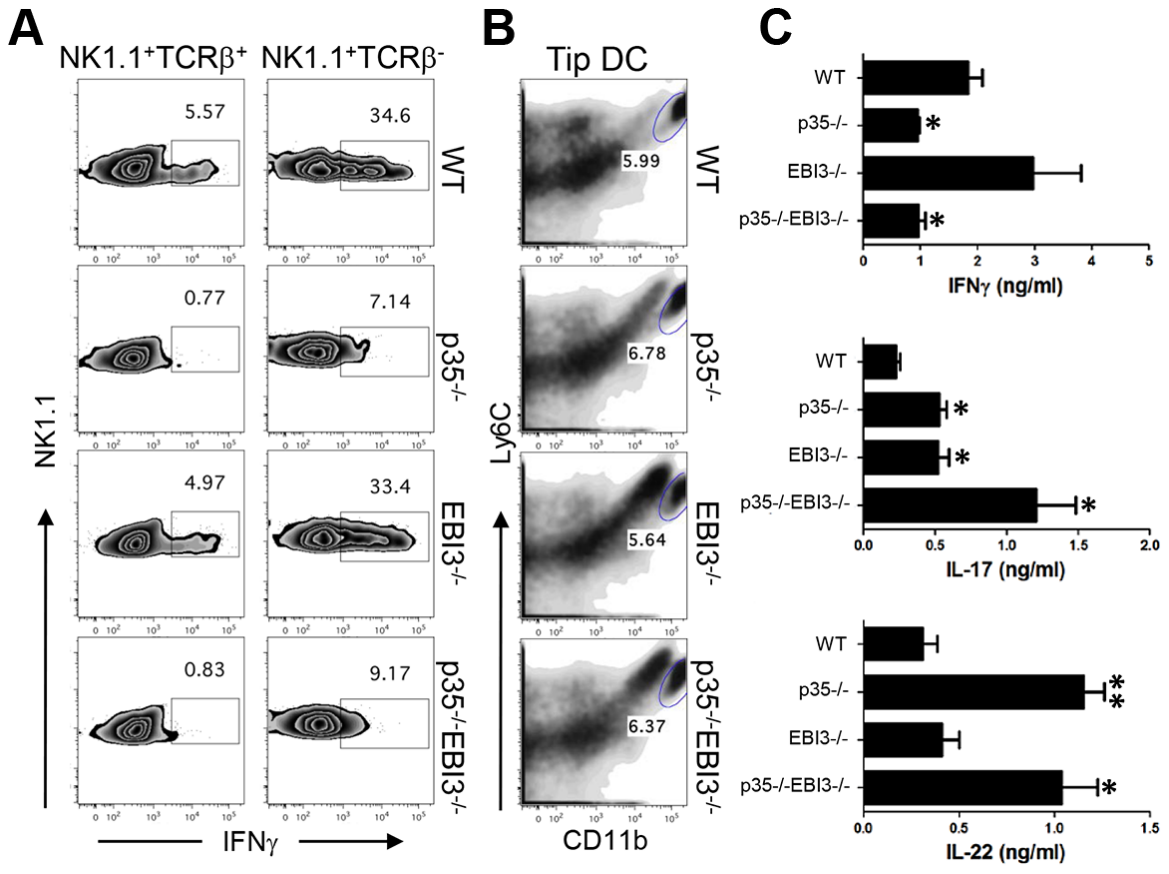
Enhanced production of Th17 cytokines in the p35^−/−^EBI3^−/−^ mice during the early phase of infection with *L. monocytogenes*. C57BL/6 (WT) or the indicated strains of mice (n = 3 per group) were intravenously infected with 2.5×10^4^ Lm-Ova on day 0. Two to three days later, lymphoid cells were analyzed for the production of IFNγ by NK and NKT cells (*A*), or for the frequency of CD11b^+^Ly6C^+^ Tip DC (*B*). The production of IFNγ, IL-17, IL-17F and IL-22 by the splenocytes obtained three days after the infection was measured (*C*). Values are mean ± SD. Data shown are representative of two independent experiments. *,p<0.05; **,p<0.01 in comparison with WT group.

To further examine the regulation of host defensive immunity by the cytokines of IL-12 family, we analyzed the activation of innate immune cells during the early phase of Lm infection. IL-12 triggers IFNγ production in NK cells and NKT cells which is critical for the activation of innate cells and the prevention of Lm propagation [28]. Consistent with this notion, we observed a significant reduction of IFNγ-producing NKT cells and NK cells in p35^−/−^ mice as well as in p35^−/−^EBI3^−/−^ mice infected with Lm-Ova (Figure 4A). The percentages of IFNγ-producing NKT cells and NK cells in EBI3^−/−^ mice were comparable to those from wild-type mice, indicating that there is no significant role of EBI3 in the induction of IFNγ from NK and NKT cells after Lm-Ova infection. Ly6C^+^CD11b^hi^ dendritic cells, also known as Tip-DC, suppress the dissemination of Lm [28], [29]. We observed comparable percentages of the Ly6C^+^CD11b^hi^ DC in p35^−/−^, EBI3^−/−^ as well as p35^−/−^ EBI3^−/−^ mice with that of wild-type mice (Figure 4B). Therefore, the induction of Tip-DC was likely normal in mice lacking p35, EBI3, or both in this experimental setting.

NK, NKT, and γδ T cells represent additional sources of innate Th1 and Th17 cytokines that could be potentially released following Lm infection. To investigate the contributions of the cellular subsets, we measured the production of IFNγ, IL-17, and IL-22 from splenocytes obtained three days after Lm-Ova infection. As depicted in Figure 4C, p35^−/−^ mice as well as p35^−/−^ EBI3^−/−^ mice showed significantly diminished IFNγ while EBI3^−/−^ mice showed comparable IFNγ production. In contrast, the amounts of IL-17 in the supernatant were higher in EBI3^−/−^ and p35^−/−^ EBI3^−/−^ mice compared with those of wild-type mice. The IL-22 production was higher in p35^−/−^ and p35^−/−^ EBI3^−/−^ mice. Collectively, these data suggest that the regulation of Th1 and Th17 cytokines by innate immune cells is also under the control of multiple IL-12 family cytokines.

### p35^−/−^EBI3^−/−^ mice are resistant to *L. monocytogenes* infection

**Figure 5:**
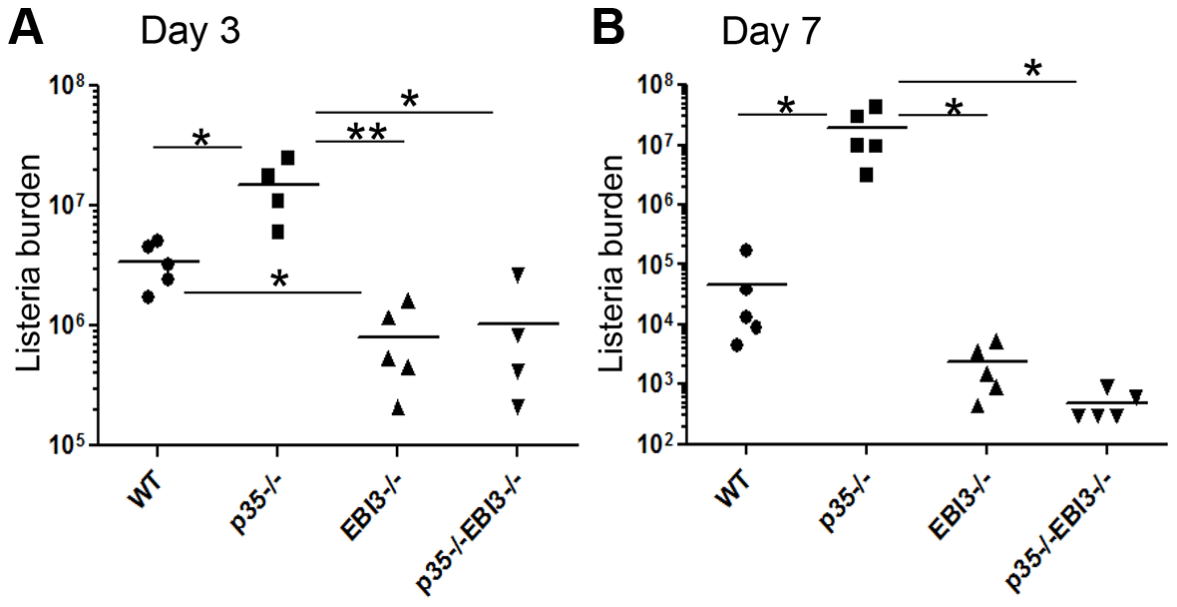
p35^−/−^EBI3^−/−^ mice are resistant to the infection with *L. monocytogenes*. C57BL/6 (WT) or the indicated strains of mice (n = 4–5 per group) were intravenously infected with 2.5×10^4^ Lm-Ova on day 0. Three (*A*) or seven (*B*) days later, the bacterial burden in the livers of the infected mice was analyzed by measuring colony-forming unit. Bars are mean values. Data shown are representative of three independent experiments. *, p<0.05 and **, p<0.01 in comparison between two indicated groups.

We next addressed the differential roles of the IL-12 family cytokines in host defense against Lm infection. Wild-type, p35^−/−^, EBI3^−/−^, or p35^−/−^EBI3^−/−^ mice were intravenously infected with Lm-Ova and bacterial burden in the livers and spleens were measured three days later. As expected, p35^−/−^ mice showed higher bacterial burden in the livers compared to wild-type controls (Figure 5A). We observed significantly less bacterial burden in the livers of EBI3^−/−^ mice compared with those from wild-type, indicating that EBI3 is not required for the host defense against the infection. To our surprise, the bacterial burden in the livers of p35^−/−^EBI3^−/−^ mice was significantly lower than that of p35^−/−^ mice, to levels comparable to EBI3^−/−^ mice (Figure 5A). Within the spleens, p35^−/−^EBI3^−/−^ mice exhibited significantly lower bacterial burden compared to p35^−/−^ mice; however, there was no evident difference in bacterial burden between wild-type and EBI3^−/−^ or p35^−/−^EBI3^−/−^ mice (Figure S1A).

We also measured bacterial burden 7 days after infection and found that p35^−/−^ mice failed to control bacterial growth with significantly higher levels of bacteria in the livers compared to wild-type animals (Figure 5B). However, EBI3^−/−^ as well as p35^−/−^ EBI3^−/−^ mice showed comparable levels of bacteria in the livers compared to wild-type mice (Figure 5B). We also observed similar pattern of bacterial burdens in the spleens of these mice (Figure S1B). Therefore, the bacterial resistance observed at day 3 largely remained intact by day 7 post infection. Collectively, these findings demonstrate that EBI3-deficiency conferred resistance to Lm-Ova infection in the absence of IL12p35, indicative of possible antagonistic function of IL-12p35 and IL-27EBI3 in host defense to the intracellular bacterial infection. Furthermore, in the absence of IL-12p35, IL-27EBI3 likely exerts strong immunosuppressive activity and thus mediates immune evasion of the Lm *in vivo*.

### IL-17 and IL-22 mediate anti-*Listeria* immunity in the absence of IL-12p35

**Figure 6:**
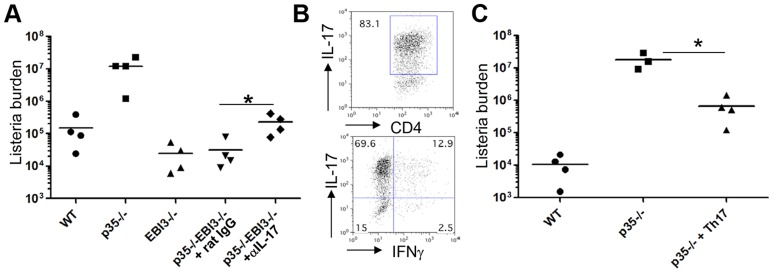
A role for IL-17 and Th17 cells on the resistance of p35^−/−^EBI3^−/−^ mice against *L. monocytogenes* infection. *A*, C57BL/6 (WT) or the indicated strains of mice (n = 4 per group) were intravenously infected with 2.5×10^4^ Lm-Ova on day 0. The mice were i.p. injected with 100 µg of anti-IL-17 or rat IgG on day 0, 2, 4. Seven days after the infection, bacterial burden in the livers of the infected mice was determined by measuring colony-forming unit. *, p<0.05 in comparison with rat IgG-treated group. *B and C*, IL-17F*^rfp^* reporter mice were s.c. immunized with LLO peptide emulsified in CFA. Seven days later, lymphoid cells from spleen and draining lymph nodes of the immunized mice were isolated and stimulated with the LLO peptide in the presence of IL-23 (50 ng/ml), IL-1β (10 ng/ml) and anti-IFNγ for 5 days. CD4^+^ RFP^+^ cells were isolated by flow cytometry, and the expression of IL-17 and IFNγ was measured by intracellular staining (*B*). The sorted Th17 cells (5×10^5^ cells per transfer) were i.v. transferred into p35^−/−^ mice, followed by i.v. infection with 2.5×10^4^ Lm-Ova. WT or p35^−/−^ mice without the cell transfer were used as controls. Seven days after infection, bacterial burden in the liver was measured (*C*). Data shown are representative of two independent experiments. *, p<0.05 in comparison with p35^−/−^ mice without Th17 cell transfer.

The enhanced production of IL-17 and IL-22 we observed in p35^−/−^EBI3^−/−^ mice by both innate and adaptive immune compartments led us to hypothesize that the induction of the Th17 cytokines might be responsible for the observed resistance of p35^−/−^EBI3^−/−^ mice against Lm-Ova infection. To test this hypothesis, we infected p35^−/−^EBI3^−/−^ mice with Lm-Ova and then injected anti-IL-17 or control Ab. Notably, the bacterial burden in the livers of the mice receiving anti-IL-17 showed a modest but significant increase (8 times higher) compared with that of the control Ab group; however the burden was still substantially lower than that observed in p35^−/−^ mice (Figure 6A). This result demonstrates that the upregulated production of IL-17, at least in part, contributed to the observed resistance of p35^−/−^EBI3^−/−^ mice to Lm-Ova infection.

Based on our findings, we hypothesized that IL-17-producing cells suppress the growth of Lm, especially in the absence of IL-12p35. To address this point, we investigated if Lm-specific Th17 cells are sufficient to limit the growth of Lm in the absence of IL-12-mediated innate and adaptive immunity. To obtain Lm-specific Th17 cells, we first isolated lymphoid cells from IL-17F*^rfp^* mice [30] after immunization with LLO_190–201_ emulsified in CFA and then restimulated them with peptide in the presence of IL-23, IL-1β and anti-IFNγ to specifically expand the Th17 population [31]. After 5 days culture, we sorted RFP^+^ CD4^+^ cells (Figure 6B; >80% IL-17^+^ and ∼15% IFNγ^+^), and transferred them i.v. into p35^−/−^ mice. Wild-type and p35^−/−^ mice receiving no cells were used as controls. All mice were then infected with Lm-Ova, and the bacterial burden in the liver was measured 7 days post infection. As shown in Figure 6C, the p35^−/−^ mice receiving the RFP^+^ CD4^+^ T cells showed significantly less bacterial load in the liver compared to p35^−/−^ mice receiving no cells (26.8 times lower), although the bacterial burden in the former group was still higher than that of the wild-type mice.

**Figure 7:**
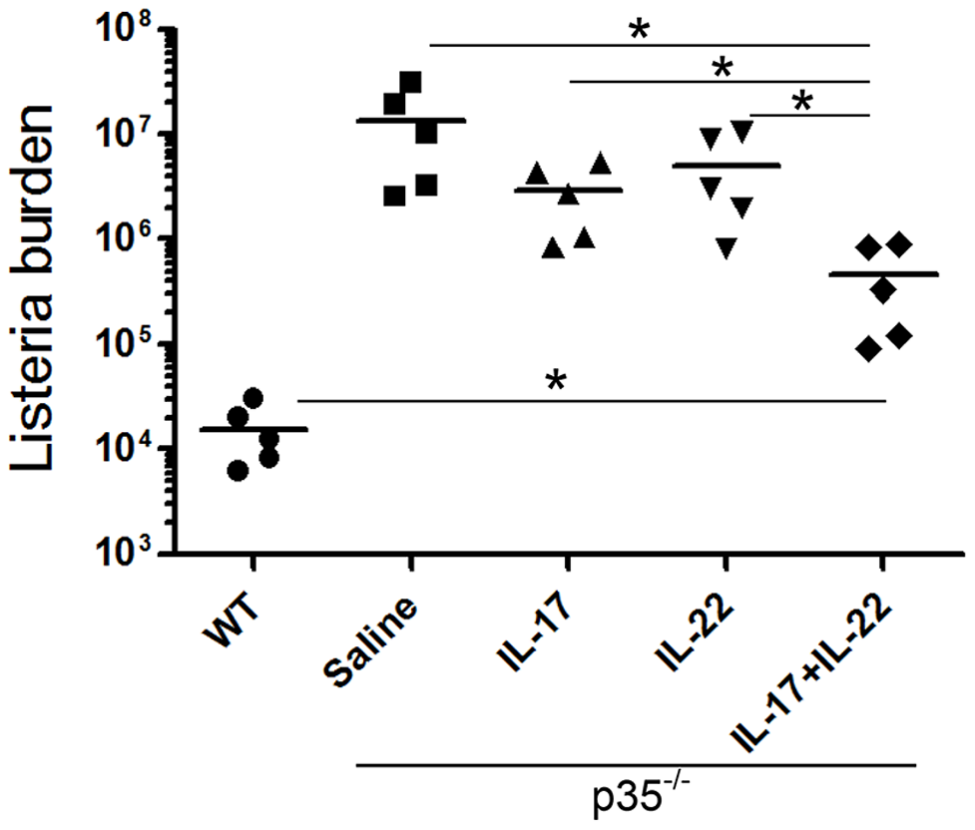
IL-17 and IL-22 cooperatively promote protective immunity against *L. monocytogenes* infection in p35^−/−^ mice. *A*, C57BL/6 (WT) or groups of p35^−/−^ mice (n = 5 per group) were intravenously infected with 2.5×10^4^ Lm-Ova on day 0. Some of the p35^−/−^ mice were i.p. injected with 1 µg of recombinant IL-17, IL-22, or both on day 0, 2, 4. Seven days after the infection, bacterial burden in the livers of the infected mice was determined by measuring colony-forming unit. Bars are mean values. *, p<0.05 in comparison between two indicated groups.

These results demonstrated that Lm-specific Th17 cells are protective against Lm-Ova in the absence of IL-12p35; however, it is possible that small population of IFNγ-producers among the RFP^+^ donor T cells (∼15%) mediated this protection. To rule out this possibility and to further determine the protective immunity mediated by IL-17 and IL-22, we next examined if administration of recombinant IL-17 or IL-22 mediates host defense against Lm-Ova in the absence of IL-12p35. As depicted in Figure 7, p35^−/−^ mice treated with IL-17 or IL-22 alone showed a slightly lower, but not statistically significant, bacterial load in the liver than saline-treated mice. Notably, administration of both cytokines induced a significantly lower bacterial burden in the liver than saline-, IL-17- or IL-22-treated p35^−/−^ mice (29.5 times less than saline-treated mice). Administration of IL-17 and IL-22, however, did not fully restore the resistance of p35^−/−^ mice, since the bacterial load was still higher than that of wild-type mice (Figure 7 and Figure S2). The inhibition of bacterial growth by exogenous IL-17 or IL-22 was more evident in the bacterial load in the spleens (Figure S2). Taken together, these results indicate that the Th17 cytokines IL-17 and IL-22 act synergistically to induce protective anti-Listeria immunity in the absence of IL-12p35.

## Discussion

In this study, we comparatively analyzed the contribution of IL-12p35 and IL-27EBI3 to the host defense against the intracellular pathogen Lm. We demonstrate that, although p35^−/−^ mice failed to control bacterial growth, mice deficient in both p35 and EBI3 had no such defect in controlling bacterial growth. Our study also revealed that IL-17 is involved in the protective immunity in p35^−/−^EBI3^−/−^ mice. Furthermore, administration of Th17 cells as well as recombinant IL-17 and IL-22 significantly suppressed bacterial growth in p35^−/−^ mice. These findings strongly suggest that Lm utilizes IL-27EBI3 to escape Th17-mediated immune surveillance in IL-12p35-deficient mice. Thus, the present study unveils a previously unappreciated immune escape mechanism of intracellular bacteria through IL-27EBI3, and that Th17 responses play an important role in intracellular bacterial infection, especially in the absence of IL-12 and Th1-mediated immunity.

NK cells, NKT cells and Tip-DC are well known innate effector cells that suppress bacterial growth during the early phase of Lm infection [28], [29]. IL-12 is required for the induction of IFNγ from NK and NKT cells which then mediates the recruitment of Tip-DC. Comparative analysis between p35^−/−^ and p35^−/−^ EBI3^−/−^ mice showed no apparent difference in the activation of NK and NKT cells and the frequency of Tip-DC. In addition, the percentages of effector CD8^+^ T cells expressing granzyme B were similar between p35^−/−^ and p35^−/−^EBI3^−/−^ mice. Moreover, although IL-27 has been reported to drive the differentiation of IL-10 producing CD4^+^ T cells [18], [19], [20], we observed comparable expression of the *Il10* transcript between wild-type and EBI3^−/−^ mice after Lm-Ova infection (data not shown). Therefore, we conclude that the increased resistance to Lm in p35^−/−^EBI3^−/−^ mice is not due to the enhanced activity of these innate immune cells nor CD8^+^ T cells.

Accumulating evidence suggests that some of the Th1 cells recruited to inflamed tissues are actually derived from Th17 cells [32], [33]. However, we observed that very few LLO-specific IFNγ-producing CD4^+^ T cells in wild-type mice after Lm infection co-expressed IL-17. In addition, LLO-specific, IFNγ-producing CD4^+^ T cells in IL-17F^Cre^×Rosa26^eYFP^ mice after Lm infection were >99% YFP-negative (data not shown), indicating that Th1 cells do not originate from Th17 cells in this model.

Notably, we observed increased production of IL-17 and IL-22 by innate immune cells, presumably γδ T cells [24], [34], as well as Lm-Ova-specific CD4^+^ T and CD8^+^ T cells in p35^−/−^EBI3^−/−^ mice. IL-22 is a Th17 cytokine that induces a series of anti-microbial peptides upon infection [4], [5], [35], [36]. The mechanism of protection by these Th17 cytokines, however, significantly differs from that of IFNγ due to the distribution of receptors and differential downstream targets. IFNγ mediates protective immunity by multiple mechanisms including the induction of iNOS and autophagy [21], [37], [38], whereas IL-17 does so possibly through neutrophil recruitment and by enhancing cross-presentation of bacterial antigens [24], [34]. In the present study, the amounts of IL-17 and IL-22 produced by innate cells and the pathogen-specific T cells were significantly increased in p35^−/−^EBI3^−/−^ mice. Furthermore, exogenous IL-17 and IL-22 synergistically induced protective immunity in p35-deficient mice, while each cytokine individually could only invoke marginal protection. Supporting this notion, it has been documented that IL-17 and IL-22 synergistically induce the expression of antimicrobial peptides [36]. Conversely, IL-22 has been shown to be dispensable for the clearance of Lm in p35-sufficient mice [39]. Our present work combined with other reports then suggests that, in the absence of IL-12-mediated protective immunity, Th17 cytokines IL-17 and IL-22 cooperatively inhibit the growth of Lm and are negatively regulated by EBI3. Importantly, since the bacterial burden in p35^−/−^ mice treated with exogenous IL-17 and IL-22 was still higher than that of wild-type mice, undefined alternative protective mechanism may still exist.

One can assume that the difference between p35^−/−^ mice and p35^−/−^EBI3^−/−^ mice in anti-Lm immunity could be due to the effect of IL-35, which is composed of p35 and EBI3 [40]. Given that p35^−/−^ mice cannot produce IL-12 and IL-35, and that p35^−/−^EBI3^−/−^ mice cannot produce IL-12, IL-35 and IL-27, the only cytokine that is lacking in the latter mice compared with the former mice is IL-27. Recent studies have shown that the other subunit of IL-27, IL-27p28, can be secreted in the absence of EBI3 to act as an antagonist of gp130 [15], [41], [42] or alternatively form a heterodimer with Cytokine-Like Factor 1 (p28/CLF) to promote NK and T cell activity [43]. Hence, EBI3-deficiency may lead to the production of p28 and p28/CLF, which may exert biological activities independently of IL-27. The role of p28 subunit of IL-27 during host defense in the present study is not clear. Future studies with p28-deficient mice will be important for a complete understanding on the mechanism by which EBI3 regulates protective immunity to intracellular pathogens.

IL-27 has been shown to trigger preliminary Th1 responses, where mice deficient in the IL-27 receptor (WSX-1^−/−^; TCCR^−/−^) are more susceptible to *Leishmania major* [12] and Lm infection [13] due to decreased Th1 responses. On the contrary, WSX-1^−/−^ mice generate more IFNγ-producing CD4^+^ T cells than wild-type mice after infection with *Toxoplasma gondii* [14], indicating that IL-27 signal is not necessary for the generation of Th1 immunity to the infection. Therefore the effect of IL-27 on pathogen-specific Th1 response is likely dependent on the infectious agents. It is not clear why IL-27EBI3^−/−^ mice in the present study did not recapitulate the phenotype of IL-27R^−/−^ mice in a previous study [13]. It is possible that the route of infection (intravenous versus subcutaneous) results in distinct immune responses to Lm. Alternatively, it is possible that the phenotype of EBI3^−/−^ mice described in this study may in fact be IL-27 independent and instead mediated through IL-27p28 [42], [44], [45]. Interestingly, fundamental differences have also been reported between WSX-1^−/−^ and EBI3^−/−^ mice. For instance, WSX-1^−/−^ mice exhibited enhanced liver inflammation, whereas EBI3^−/−^ mice showed reduced liver inflammation in the same Con A-induced hepatitis animal model [46], [47]. Moreover, while T cells from WSX-1^−/−^ mice produce less IFNγ, T cells from EBI3^−/−^ mice produce higher IFNγ and less IL-4 than wild-type T cells [12], [13], [48]. Further study is needed to demonstrate the mechanism of these differences in the regulation of infectious and inflammatory diseases between the EBI3 and IL-27 receptor signaling pathways.

Collectively our findings demonstrate that the immune system produces IL-12 to suppress bacterial growth upon infection while Lm utilizes another host immune component, EBI3, to escape immune surveillance. Increased susceptibility to intracellular pathogens in patients with deficiency in IL-12 or its receptor has been demonstrated [49], [50]. Based on our findings, blockade of EBI3 may provide a new therapeutic approach for the treatment of infectious diseases, particularly in patients with defective IL-12 immunity.

## Materials and Methods

### Ethics statement

All the animal experiments were performed in accordance with the Guide for the Care and Use of Laboratory Animals of the National Institutes of Health and with the permission of the American Association for the Assessment and Accreditation of Laboratory Animal Care. The protocol was reviewed and approved by the Institutional Animal Care and Use Committee of MD Anderson Cancer Center (identification number: 10-04-09833) and University of Texas Health Science Center at Houston (identification number: HSC-AWC-12-008).

### Mice

C57BL/6 and IL-12p35^−/−^ mice were purchased from the Jackson Laboratory. IL-27EBI3^−/−^ mice were generated as described previously [48]. Double-deficient mice (p35^−/−^EBI3^−/−^) were obtained by crossing IL-12p35^−/−^ and IL-27EBI3^−/−^ mice. IL-17F*^rfp^*-reporter mice were generated as described previously [30]. All mice were kept under specific pathogens-free condition. The animal experiments were performed at the age of 6–12 weeks.

### Stimulation of bone marrow-derived dendritic cells (BM-DC) and macrophages (BM-M)

Bone marrow cells from femurs and tibia of C57BL/6 mice were cultured with 10% FBS supplemented RPMI containing GM-CSF or M-CSF for 6 days. For irradiation, log-phase cultured Lm-Ova were exposed to 300 K rad of γ-irradiation. After extensive washing, BM-DC and BM-M cells were incubated with the irradiated Lm-Ova at the ratio of 1∶10. As controls, LPS (100 ng/ml) and Pam3CysSK4 (Pam; 1 µg/ml) were added in the culture. Four hours after the stimulation, cells were harvested and resuspended in Trizol for mRNA expression analysis.

### Infection with Listeria monocytogenes

An erythromycin resistant strain of Lm-Ova was grown in brain heart infusion media supplemented with 5 µg/ml erythromycin [51]. The bacteria were harvested at mid-log growth phase and were intravenously injected into animals (2.5×10^4^ CFU/mouse). In some experiments, mice were intraperitoneally administered recombinant murine IL-17, IL-22 (Peprotech), or both (1 µg/injection) on day 0, 2, 4 after infection. Three or seven days after infection, spleens and livers of the infected mice were harvested. Bacterial burdens were determined by measuring colony forming unit, as described previously [52]. Splenocytes were stimulated with SIINFEKL peptide or LLO_190–201_ peptide overnight for intracellular cytokine staining, or 3 days for ELISA analysis [52]. In some experiments, splenocytes were resuspended in Trizol for mRNA expression analysis.

### Antibodies used for flow cytometric analysis

The following antibodies were used for cell surface and intracellular staining; PerCPCy5-5- or FICT-labeled anti-TCRβ (H57-597), PerCPCy5-5-labeled anti-CD4 (GK1.5), Alexa 488-labeled anti-CD8 (5H10-1), APC-labeled anti-CD11b (M1/70) from Biolegnd; PE- or Alexa 488-labeled anti-IFNγ (XMG1.2), PE-labeled anti-IL-17 (clone TC11-18H10), Alexa 647-labeled anti-GranzymeB (GB11) FITC- or PerCPCy5.5-labeled anti-NK1.1 (PK136), PerCPCy5-5-labeled anti-Ly6C (AL21) from BD Biosciences. For intracellular staining, cells were incubated with permeabilization buffer (BD Biosciences), and then further stained with intracellular staining Abs described above. These cells were analyzed by using LSRII flow cytometer (BD Bioscience) and Flowjo software.

### Real-time PCR analysis for mRNA expression

Total RNA was prepared from splenocytes with TriZol reagent (Invitrogen). Complementary DNA (cDNA) was synthesized with Superscript reverse transcriptase and oligo(dT) primers (Invitrogen), and gene expression was examined with a Bio-Rad iCycler Optical System with iQ SYBR green real-time PCR kit (Bio-Rad Laboratories). The data were normalized to *Actb* reference. The following primer pairs were used: *ActB*: F-GAC GGC CAG GTC ATC ACT ATT G and R-AGG AAG GCT GGA AAA GAG CC; *Ifng*: F-GAT GCA TTC ATG AGT ATT GCC AAG T and R-GTG GAC CAC TCG GAT GAG CTC; *Il17*: F-CTG GAG GAT AAC ACT GTG AGA GT and R-TGC TGA ATG GCG ACG GAG TTC; *Il17f*: F-CTG GAG GAT AAC ACT GTG AGA GT-3′ and R-TGC TGA ATG GCG ACG GAG TTC; *Il22*: F-CAT GCA GGA GGT GGT ACC TT and R-CAG ACG CAA GCA TTT CTC AG; *Il10*: F-ATA ACT GCA CCC ACT TCC CAG TC and R-CCC AAG TAA CCC TTA AAG TCC TGC; *Ebi3*: F-TCC CCG AGG TGC AAC TGT TCT CC and R-GGT CCT GAG CTG ACA CCT GG. Primers for p35, p40, p19 were described previously [53].

### Adoptive transfer study

To obtain IL-17-producing CD4^+^ T cells specific for Lm-Ova, we s.c. immunized IL-17F*^rfp^*-reporter mice with LLO peptide in CFA. A week later, lymphoid cells from the draining lymph nodes and spleen were pooled and restimulated with the same peptide in the presence of IL-23 (50 ng/ml) and IL-1β (10 ng/ml) plus anti-IFNγ (5 µg/ml; XMG1.2) for five days. The cells were stained with APC-labeled anti-CD4, and APC-positive and RFP-positive cells were sorted by using FACS-Influx (BD Biosciences). 2.5×10^5^ sorted cells/mouse were intravenously injected into IL-12p35^−/−^ mice followed by Lm-Ova inoculation and analysis of bacterial burden, as described above.

### Statistical analysis

The Student *t* test was used to assess the statistical values. *P* values were determined, and error bars represent standard error of the mean (SEM) or standard deviation (SD).

## Supporting Information

Figure S1 **Bacterial load in the spleens of p35^−/−^ and EBI3^−/−^ mice after infection with** ***L. monocytogenes*** **.** C57BL/6 (WT) or the indicated strains of mice (n = 4–5 per group) were intravenously infected with 2.5×10^4^ Lm-Ova on day 0. Three (*A*) or seven (*B*) days later, the bacterial burden in the spleens of the infected mice was analyzed by measuring colony-forming unit. Bars are mean values. Data shown are representative of three independent experiments. *, p<0.05 and **, p<0.01 in comparison between two indicated groups.
(PDF)

Figure S2 **Bacterial load in the spleens of p35^−/−^ mice treated with IL-17 or IL-22.** *A*, C57BL/6 (WT) or groups of p35^−/−^ mice (n = 5 per group) were intravenously infected with 2.5×10^4^ Lm-Ova on day 0. Some of the p35^−/−^ mice were i.p. injected with 1 µg of recombinant IL-17, IL-22, or both on day 0, 2, 4. Seven days after the infection, bacterial burden in the spleens of the infected mice was determined by measuring colony-forming unit. Bars are mean values. *, p<0.05 in comparison between two indicated groups.
(PDF)
