# Supplementary figures and images for: Epstein Barr Virus-Induced 3 (EBI3) Together with IL-12 Negatively Regulates T Helper 17-Mediated Immunity to Listeria monocytogenes Infection
Source: PLoS Pathog. 2013 Sep 19;9(9):e1003628. doi: 10.1371/journal.ppat.1003628 (PMC3777861; doi:10.1371/journal.ppat.1003628)

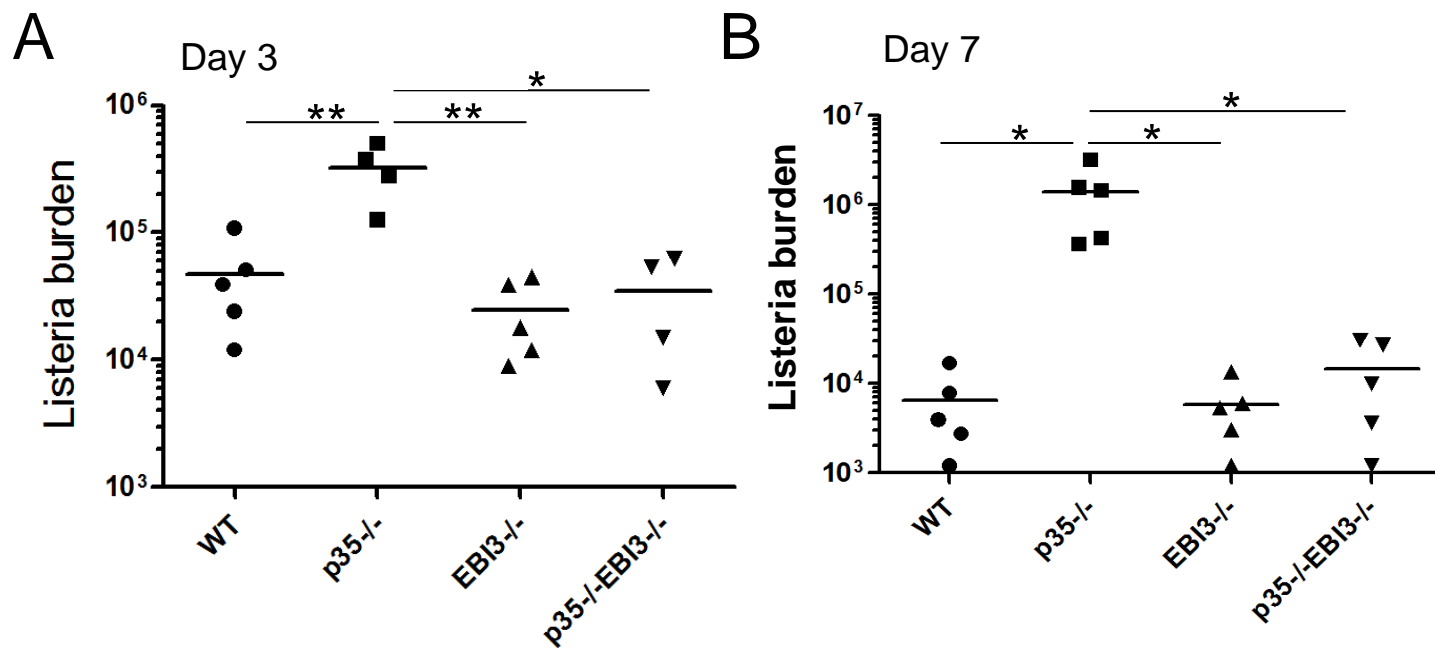

Figure S1.

Supplement: Figure S1 — Bacterial load in the spleens of p35−/− and EBI3−/− mice after infection with L. monocytogenes . C57BL/6 (WT) or the indicated strains of mice (n = 4–5 per group) were intravenously infected with 2.5×10^4^ Lm-Ova on day 0. Three (A) or seven (B) days later, the bacterial burden in the spleens of the infected mice was analyzed by measuring colony-forming unit. Bars are mean values. Data shown are representative of three independent experiments. *, p<0.05 and **, p<0.01 in comparison between two indicated groups. (PDF) [file ppat.1003628.s001.pdf]

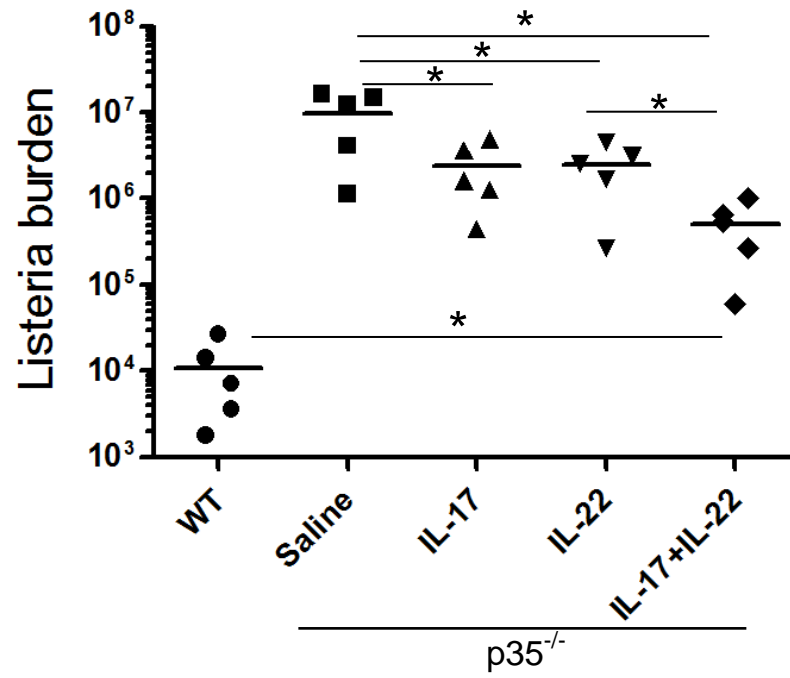

Figure S2.

Supplement: Figure S2 — Bacterial load in the spleens of p35−/− mice treated with IL-17 or IL-22. A, C57BL/6 (WT) or groups of p35−/− mice (n = 5 per group) were intravenously infected with 2.5×10^4^ Lm-Ova on day 0. Some of the p35−/− mice were i.p. injected with 1 µg of recombinant IL-17, IL-22, or both on day 0, 2, 4. Seven days after the infection, bacterial burden in the spleens of the infected mice was determined by measuring colony-forming unit. Bars are mean values. *, p<0.05 in comparison between two indicated groups. (PDF) [file ppat.1003628.s002.pdf]
